# The Significance of HIV ‘Blips’ in Resource-Limited Settings: Is It the Same? Analysis of the Treat Asia HIV Observational Database (TAHOD) and the Australian HIV Observational Database (AHOD)

**DOI:** 10.1371/journal.pone.0086122

**Published:** 2014-02-07

**Authors:** Rupa Kanapathipillai, Hamish McManus, Adeeba Kamarulzaman, Poh Lian Lim, David J. Templeton, Matthew Law, Ian Woolley

**Affiliations:** 1 Monash Medical Centre, Clayton, Victoria, Australia; 2 Alfred Hospital, Prahran, Victoria, Australia; 3 Kirby Institute, The University of New South Wales, Darlinghurst, New South Wales, Australia; 4 University of Malaya Medical Centre, Kuala Lumpur, Malaysia; 5 Department of Infectious Diseases, Tan Tock Seng Hospital, Singapore; 6 RPA Sexual Health, Royal Prince Alfred Hospital, Sydney, New South Wales, Australia; 7 Departments of Medicine and Infectious Diseases, Monash University, Clayton, Victoria, Australia; University of Pittsburgh, United States of America

## Abstract

**Introduction:**

Magnitude and frequency of HIV viral load blips in resource-limited settings, has not previously been assessed. This study was undertaken in a cohort from a high income country (Australia) known as AHOD (Australian HIV Observational Database) and another cohort from a mixture of Asian countries of varying national income per capita, TAHOD (TREAT Asia HIV Observational Database).

**Methods:**

Blips were defined as detectable VL (≥ 50 copies/mL) preceded and followed by undetectable VL (<50 copies/mL). Virological failure (VF) was defined as two consecutive VL ≥50 copies/ml. Cox proportional hazard models of time to first VF after entry, were developed.

**Results:**

5040 patients (AHOD n = 2597 and TAHOD n = 2521) were included; 910 (18%) of patients experienced blips. 744 (21%) and 166 (11%) of high- and middle/low-income participants, respectively, experienced blips ever. 711 (14%) experienced blips prior to virological failure. 559 (16%) and 152 (10%) of high- and middle/low-income participants, respectively, experienced blips prior to virological failure. VL testing occurred at a median frequency of 175 and 91 days in middle/low- and high-income sites, respectively. Longer time to VF occurred in middle/low income sites, compared with high-income sites (adjusted hazards ratio (AHR) 0.41; p<0.001), adjusted for year of first cART, Hepatitis C co-infection, cART regimen, and prior blips. Prior blips were not a significant predictor of VF in univariate analysis (AHR 0.97, p = 0.82). Differing magnitudes of blips were not significant in univariate analyses as predictors of virological failure (p = 0.360 for blip 50–≤1000, p = 0.309 for blip 50–≤400 and p = 0.300 for blip 50–≤200). 209 of 866 (24%) patients were switched to an alternate regimen in the setting of a blip.

**Conclusion:**

Despite a lower proportion of blips occurring in low/middle-income settings, no significant difference was found between settings. Nonetheless, a substantial number of participants were switched to alternative regimens in the setting of blips.

## Background

A number of studies have addressed the long-term significance of viral “blips” in the setting of treated HIV infection [1]–[9]. The definition of a blip has evolved over the last decade and now is defined as ‘after virological suppression, an isolated detectable HIV RNA level followed by return to virological suppression [10]. Though studies have differed in their definition of blips and virological failure/rebound, the majority of studies demonstrate no association between the occurrence of blips *per se* and development of virological failure [2]–[6], [11]–[15]. Few studies have demonstrated an increased risk of virological failure[1], [8], [16]–[18]. However, the magnitude of blips has been found to be associated with increased risk of virological failure/rebound [11], [17] with a recent study demonstrating a significantly higher risk with blips >500 copies/ml [16].

The true aetiology of blips remains uncertain. One or a combination of causal factors have been suggested including random biological fluctuation and statistical variation [4], release of virus from latent reservoirs [19], intercurrent infection [20], laboratory collection and processing [21], and differing sensitivities of respective assays [4], [22], [23], especially at low levels of viremia [24].

In addition it is possible that blips in the setting of drugs with a low genetic barrier to resistance, such as NNRTI, may have different impact on subsequent risk of VF compared with other classes of drug such as protease inhibitors or integrase inhibitors. Few studies have formally assessed the role of blips on virological outcomes between classes of antiretrovirals [12], [15], [18].

Blips could have different significance in settings where HIV virological monitoring occurs less frequently, and because of resource limitations blips may be managed differently in resource-limited, compared with resource-rich, settings. In addition, less frequent virological monitoring in resource-poor settings may result in different interpretations of blips when they are detected. To our knowledge there have been no previous studies examining the significance of blips in resource poor settings.

The primary objective of this study was to compare the significance of blips in resource-poor and resource-replete settings. Secondary objectives were to assess the significance of differing magnitudes and frequency of blips in a given year, and the significance of blips with differing definitions of virological failure.

### Study Design and Cohort Description

This was an analysis of patients from the Treat Asia HIV Observational Database (TAHOD) and Australian HIV Observational Database (AHOD). TAHOD is an observational cohort of 17 low- middle- and high-income clinical sites in the Asia and Pacific region, specifically Cambodia, China, Hong Kong, India, Indonesia, Japan, Malaysia, the Philippines, Singapore, South Korea, Taiwan, and Thailand [25] ^25^. Sites are stratified into low-, middle- and high-income based on gross national income per capita [26]. AHOD is comprised of 27 high-income clinical sites throughout Australia [27]. HIV infected patients, aged >18 years, from TAHOD and AHOD who had documented VL <50 after commencement of cART, prior to 31 March 2011 were included in this study. The earliest recorded date of commencement of cART was 23 August 1996 and 1 June 1998 for AHOD and TAHOD respectively. Study follow-up was to 31 March 2011. All patients in TAHOD and AHOD have baseline CD4 and HIV viral load following diagnosis, with subsequent immunological and virological monitoring frequency varying from site to site. Only patients with recorded undetectable HIV viral load at or after commencement of cART were included in the analysis. Recorded CD4 within 180 days prior to first cART and HIV viral load within 360 days prior to first cART were used.

Patients were commenced on cART, with a minimum of 3 antiretrovirals, in accordance with national guidelines and clinician discretion. Core data variables recorded in TAHOD and AHOD include: gender; date of birth; date of most recent visit; HIV exposure; CD4 and CD8 cell counts; HIV viral load; antiretroviral treatment data; AIDS-defining illnesses; hepatitis B virus (HBV) surface antigen status; hepatitis C virus (HCV) antibody status; and date and cause of death.

Patient selection and extraction of data occur at data centres of the participating sites. Written informed consent is obtained from all patients at the time of enrollment. TAHOD and AHOD data is aggregated at The Kirby Institute, University of New South Wales. Ethical approval for the study was obtained from the University of New South Wales Ethics Committee. Each site also obtained approval from their local ethics committee. As part of the approved AHOD protocol, all data is formally owned by the contributing clinical sites, and is collated on their behalf at The Kirby Institute. Data is transferred to the Kirby Institute in a de-identified format. Data is stored in a combined format on The Kirby Institute local area network, a password protected system, with access restricted to the study’s Research Coordinators and Statisticians. Data may be released to researchers on application in a de-identified format following local ethics approval.

## Statistical Methods

Blips were defined as detectable viral load (≥50 copies/ml) preceded and followed by undetectable viral load (<50 copies/mL). Episodes of persistently detectable, defined as two or more consecutive viral loads ≥50 copies/mL, were not considered blips. “Real” blips were those blips that did not involve a change in therapy as a result of a VL>50 copies/ml. “Switched blips” were defined as undetectable viral load followed by a single elevated VL followed by a change in therapy. Virological failure was defined as two consecutive viral load ≥50 copies/ml after viral suppression post commencement of cART. Only data available from the time of each site having a “sensitive” viral load assay which could read down to 50 copies was included in the study so biases due to different types of assay reading down to 400 copies rather than 50 were reduced. Instances of subsequent intermittent tests at thresholds of less sensitive assays were excluded from the analysis.

Based on survival models, the covariate ‘Year of first cART’ was dichotomized to before or after 2004, with equal numbers commencing cART either before or after this date. Subsequent inclusion into the model was based at time of virological control. Cox models of time to first virological failure after entry, following control of viral load after commencement of cART, were developed. Patients were censored at death, or the earlier of lost to follow up, 180 days after last viral measure, or 31 March 2011. Periods off treatment of greater than 14 days were excluded from models. Intervals of blip duration were excluded. Durations of blip, were excluded from analysis follow-up time. Multivariate models were developed using forward stepwise selection from significant univariate predictors (p<0.05) with forced inclusion of income level and prior blips. Low- and Middle-income site data was amalgamated because of insufficient observations in the low-income category. Sensitivity analysis of differing definitions of virological failure were conducted, specifically virological failure defined as 2 consecutive viral loads of ≥200 copies/mL, ≥400 copies/mL, and ≥1000 copies/mL. Sensitivity analyses were performed to assess the significance of varying magnitudes of blips, specifically to viral load thresholds of 200, 400 and 1000 copies/mL. A sensitivity analysis of ‘switched blips’ as determinants of virological failure was conducted.

## Results

A total of 5040 patients, 2597 from AHOD and 2521 from TAHOD were included. 4167 (82.7%) participants were male, mean CD4 at cART initiation was 294.4 and 152.5 cells/mm^3^ in high- and middle/low-income settings, respectively. Viral load testing occurred at a median of 175 and 91 days in low/medium-, and high-income sites respectively. The median duration of follow-up between viral control after first cART to censor was 3.20 years [IQR 1.36–6.13]. Other patient demographic and clinical characteristics are outlined in Table 1.

**Table 1:** Patient characteristics.

	HIC (%)	M/LIC (%)
**N**	3,486	1,554
**Gender**		
Male	3,250 (93)	917 (59)
Female	236 (7)	637 (41)
**Exposure**		
MSM	2,382 (68)	108 (7)
IDU	148 (4)	72 (5)
Heterosexual	588 (17)	1,262 (81)
Other	346 (10)	101 (6)
Missing	22 (1)	11 (1)
**Year of first cART**		
<2004	2,380 (68)	781 (50)
≥2004	1,106 (32)	773 (50)
**ADI**		
No	2,646 (76)	858 (55)
Yes	840 (24)	696 (45)
**HCV ever**		
No	2,794 (80)	830 (53)
Yes	308 (9)	138 (9)
Missing	384 (11)	586 (38)
**HBV ever**		
No	2,763 (79)	929 (60)
Yes	196 (6)	116 (7)
Missing	527 (15)	509 (33)
**CD4 at First cART**		
Mean (SD)	294.4 (225.63)	152.5 (166.87)
<200	1039 (30)	910 (59)
200–349	839 (24)	240 (15)
350–499	494 (14)	60 (4)
≥500	436 (13)	52 (3)
Missing	678 (19)	292 (19)
**Viral load at first cART**		
Median (IQR)	52,930 (9,316–177,091.5)	62,143.5 (5,980–272,000)
≤50	90 (3)	85 (5)
51–1,000	226 (6)	41 (3)
1,001–10,000	372 (11)	63 (4)
>10,000	1,911 (55)	465 (30)
Missing	887 (25)	900 (58)
**Age at first cART**		
Mean (SD)	39.8 (10.22)	36.1 (9.01)
<30	524 (15)	375 (24)
30–39	1,365 (39)	727 (47)
40–49	1,006 (29)	313 (20)
50–59	439 (13)	105 (7)
60–69	152 (4)	34 (2)

cART - combination antiretroviral therapy.

HIC - High Income Countries.

M/LIC - Middle/Low Income Countries.

ADI - AIDS Defining Illnesses.

HCV - Hepatitis C Virus.

HBV - Hepatitis B Virus.

Virological failure (following virological control post commencement of cART) occurred in 1037 patients (20.6%) at a median of 1.63 years (IQR: 0.87–3.23) and 1.71 years (IQR 0.74–3.12) post commencement of cART in high- and low/middle- income sites respectively.

Hepatitis C antibody positivity was associated with a shorter time to virological failure. cART containing NRTI+PI), NNRTI+PI±NRTI, 3+NRTI/no PI/no NNRTI HR, and other regimens were associated with significantly shorter time to virological failure, compared to the most frequently used first-line regimen (3+NRTI+NNRTI/no PI). Commencement of cART after 2004, and heterosexual exposure compared with MSM, was associated with significantly longer time to virological failure. Income was associated with significantly reduced time to virological failure in multivariate analysis adjusted for all covariates as outlined in Table 2.

**Table 2:** Cox proportional hazards models of time until first viral failure after entry.

	Univariate		Multivariate	
	HR (95% CI)	(p-val)	HR (95% CI)	(p-val)
**Income**				
Medium/Low (ref (High)	0.32 (0.26–0.38)	(<0.001)	0.42 (0.34–0.51)	<0.001
**Age at first cART**				
−29	0.93 (0.77–1.13)	(0.476)		
30–39	1.09 (0.93–1.26)	(0.28)		
40–49 (reference)	1			
50–59	1.02 (0.82–1.27)	(0.858)		
60-	1 (0.7–1.43)	(0.999)		
**Gender**				
Female (ref Male)	0.46 (0.38–0.57)	(<0.001)		
**Year of first cART**				
> = 2004 (ref(<2004)	0.29 (0.24–0.35)	(<0.001)		
**Exposure**				
IDU (ref MSM)	1.23 (0.94–1.62)	(0.131)		
HET	0.47 (0.4–0.54)	(<0.001)		
OTHER	0.81 (0.65–1.02)	(0.068)		
MISSING	0.59 (0.22–1.59)	(0.3)		
**CD4 at first cART**				
200–349 (ref −199)	1.11 (0.94–1.31)	(0.202)		
350–499	1.12 (0.92–1.38)	(0.263)		
500-	1.3 (1.05–1.6)	(0.015)		
Missing	1.04 (0.87–1.23)	(0.678)		
**Viral load at first cART**				
51–1000 (ref undetectable)	3.68 (2.04–6.65)	(<0.001)		
1001–10000	3.11 (1.74–5.54)	(<0.001)		
10001-	3.22 (1.86–5.58)	(<0.001)		
Missing	2.56 (1.47–4.46)	(0.001)		
**AIDS^1^**				
Yes (ref No)	0.94 (0.83–1.07)	(0.364)		
**HCV ever**				
Yes (ref No)	1.41 (1.16–1.72)	(0.001)	1.35 (1.11–1.64)	0.003
Missing	0.87 (0.73–1.04)	(0.117)	1.21 (1.01–1.45)	0.037
**HBV ever**				
Yes (ref No)	0.94 (0.73–1.21)	(0.622)		
Missing	0.83 (0.7–0.98)	(0.032)		
**Regimen^1^**				
3+ NRTI+PI,NO NNRTI,NO II (ref 3+ NRTI+NNRTI,NO PI,NO II)	1.52 (0.95–2.44)	(0.083)	1.31 (0.82–2.11)	0.263
3+ NNRTI+PI,+/−NRTI,NO II	2.46 (1.93–3.14)	(0)	1.61 (1.25–2.06)	<0.001
3+ NRTI,No PI,NO NNRTI,NO II	1.97 (1.73–2.26)	(0)	0.34 (0.28–0.41)	<0.001
3+ II,+/−NRTI,+/−NNRTI,+/−PI	3.43 (2.69–4.39)	(0)	2.22 (1.73–2.85)	<0.001
**Prior blip^1^**				
Yes (ref No)	0.97 (0.77–1.23)	(0.82)	0.82 (0.65–1.03)	0.092

Sensitivity analysis assessing differing definitions of virological failure showed that income remained a significant predictor of time to virological failure for VL ≥200 copies/mL (HR 0.35 (95% CI 0.28–0.43) p<0.001) comparing low/middle-income sites to high-income sites, for VL ≥400 copies/mL (HR 0.37 (95% CI 0.29–0.46) p<0.001)and for VF ≥1000 copies/mL (HR 0.0.36 (95% CI 0.28–0.47) p = 0.07). Prior blips were not significant with these differing definitions of virological failure; VF ≥200 copies/mL HR 0.92 (95% CI 0.69–1.23) p = 0.57, VF ≥400 copies/mL HR 0.94 (95% CI 0.69–1.27) p = 0.67, VF ≥1000 copies/mL HR 0.1.00 (95% CI 0.72–1.39), p = 0.98.

Nine hundred and ten (18%) of patients experienced blips ever (both before and after episodes of viral failure following cART). 744 (21%) and 166 (11%) of high- and middle/low-income participants, respectively, experienced blips ever. Seven hundred and eleven (14%) of all patients experienced blips prior to the earlier of censoring or virological failure. 559 (16%) and 152 (10%) of high- and middle/low-income participants, respectively, experienced blips. The overall rate of blips was 50.3 (95% CI: 46.7–54.1), and 31.7 (95% CI: 27.4–36.6) per 1000 person years of follow-up in high- and middle/low-income sites, respectively. However, the ratio of blips to number of viral load tests performed was similar across sites; 1.47% and 1.64% in high- and low/middle-income sites respectively.

Testing frequency was not adjusted for in the primary analysis. A sensitivity analysis adjusting for testing frequency showed no qualitative difference in results. A sensitivity analysis to assess the significance of blips allowing for different testing intervals was conducted by using every second test result from HIC. This corresponded to a biannual testing frequency equivalent to the testing frequency of L/MIC. A separate sensitivity analysis using entry after more than one VL<50 copies/mL after first cART is qualitatively the same as primary analysis.

572 (63%) of all blips were between 50–199 copies/mL, 114 (13%) were between 200–399 copies/mL, 98 (11%) were between 400–1000 copies/mL and 126 (14%) were greater than 1000 copies/ml. Differing magnitudes of blips were not significant in univariate analyses as predictors of virological failure (p = 0.360 for blip 50–≤1000, p = 0.309 for blip 50–≤400 and p = 0.300 for blip 50-≤200). Differing frequencies of blips, compared to no blips, were not significant predictors of virological failure in univariate analysis; single blip HR 0.96 (95% CI 0.75–1.22); multiple blips HR 1.19 (95% CI 0.59–2.41).

209 of 866 (24%) patients were switched to an alternate regimen in the setting of a single low viral load reading, thus meeting our definition of a ‘switched blip’. The median viral loads of patients that triggered this switch were 285.5 copies/mL (IQR 89–1000), and 257 copies (IQR 72–757) for high-, and low/middle-income sites respectively. There was no significant increase in time to virological failure with ‘switched’ blips HR = 1.10 (95% CI 0.91–1.33), p = 0.33). 63 (30%) of ‘switched blips’ occurred in middle/low-income sites. Most ‘switched blips’ occurred in high-income settings and before 2006 (68%). Most were switched from NRTI+PI (48%) regimens; of patients with ‘switched blips’, the majority was switched to NRTI+PI (46%) or NRTI+NNRTI (45%) regimens.

## Discussion

Our study demonstrates that the presence of blips, irrespective of magnitude or frequency, is not predictive of virological failure. This finding also holds true in low/middle- income countries, where viral load testing is much less frequent than in high-income countries. Our study, additionally, demonstrates that a large proportion of patients undergo switching of antiretroviral regimen in the setting of blips.

Our study findings are in keeping with previous studies, conducted only in resource-rich settings, that found no association between blips and virological failure [2]–[6], [11]–[15]. Two hundred and nine patients were switched to a different cART regimen in the setting of a blip. The findings of this study suggest that many of those switches may have been made unnecessarily. In resource-limited settings, with limited antiretroviral options, these unnecessary switches may have significant implications in terms of cost and pill-burden [28]. The majority of switches occurred in resource-rich settings, perhaps reflecting an ongoing degree of clinical uncertainty about the significance of blips.

A major strength of our study is that, to our knowledge, it is the first to assess the significance of blips in resource-limited settings. It reflects ‘real-world’ decision-making and outcomes, and has identified a sizeable proportion of patients who have potentially undergone unnecessary switching of cART in the setting of blips. While it does not obviate the need for viral load testing in resource-poor settings, it provides reassuring outcomes regarding infrequent viral load testing in this very specific setting of blips. Our study is also the first to address the significance of blips with differing definitions of virological failure – relevant in resource-limited settings, which often have higher thresholds for defining virological failure.

Our study has several limitations. Analysis of duration of specific antiretroviral regimens, and switch to therapy prior to the development of virological failure were not included in this analysis. Regimens varied widely between sites and have changed significantly over the treatment duration included in this analysis, including triple NRTI regimens and un-boosted protease inhibitors. This analysis did not include details of the different viral load assays used at various sites at different times. Viral load assays are known to have different sensitivities [4], [22]–[24]. The significance of varying sensitivities of viral load assays is unknown in this assessment, but again, reflects ‘real-world’ decision-making as knowledge of differing assays used is unlikely to have changed clinical practice. No data on adherence was able to be included in this analysis.

This study demonstrates that frequency of viral load testing varies enormously between sites. Despite this, no significant increased risk of virological failure was demonstrated in the setting of blips. Questions remain about the significance of more prolonged low-level viremia in settings with infrequent viral load testing that warrant further investigation.
